# Comparing Clinical Outcomes on Oncology Patients With Severe Aortic Stenosis Undergoing Transcatheter Aortic Valve Implantation: A Systematic Review and Meta-Analysis

**DOI:** 10.3389/fcvm.2022.890082

**Published:** 2022-05-31

**Authors:** Yumeng Song, Yutong Wang, Zuoxiang Wang, Chang Xu, Jingshen Dou, Tingbo Jiang

**Affiliations:** ^1^Department of Cardiology, The First Affiliated Hospital of Soochow University, Suzhou, China; ^2^Department of Medicine, Soochow University, Suzhou, China

**Keywords:** aortic stenosis, oncology, transcatheter aortic valve implantation, meta-analysis, mortality

## Abstract

**Objective:**

To compare the clinical outcomes of cancer and non-cancer patients with severe aortic stenosis (AS) after transcatheter aortic valve implantation (TAVI).

**Methods:**

A computer-based search in PubMed, EMbase, The Cochrane Library, CBM, CNKI, and Wanfang databases from their date of inception to October 2021, together with reference screening, was performed to identify eligible clinical trials. Two reviewers independently screened the articles, extracted data, and evaluated their quality. Review Manger 5.3 and Stata 12.0 software were used for meta-analysis.

**Results:**

The selected 11 cohort studies contained 182,645 patients, including 36,283 patients with cancer and 146,362 patients without cancer. The results of the meta-analysis showed that the 30-day mortality [*OR* = 0.68, 95%*CI* (0.63,0.74), *I*^2^= 0, *P* < 0.00001] of patients with cancer in the AS group was lower than those in the non-cancer group; 1-year mortality [*OR* = 1.49, 95%*CI*(1.19,1.88), *I*^2^= 58%, *P* = 0.0006] and late mortality [*OR* = 1.52, 95%*CI*(1.26,1.84), *I*^2^= 55%, *P* < 0.0001] of patients with cancer in the AS group was higher than those in the non-cancer group. The results of the meta-analysis showed that the stroke [*OR* = 0.77, 95%*CI* (0.72, 0.82), *I*^2^= 0, *P* < 0.00001] and the acute kidney injury [*OR* = 0.78, 95%*CI* (0.68, 0.90), *I*^2^= 77%, *P* = 0.0005] of patients with cancer in the AS group was lower than those in the non-cancer group. The results of the meta-analysis showed no statistical difference in cardiovascular mortality, bleeding events, myocardial infarction, vascular complication, and device success rate.

**Conclusion:**

It is more effective and safer in patients with cancer with severe AS who were undergoing TAVI. However, compared with patients with no cancer, this is still high in terms of long-term mortality, and further study of the role of TAVI in patients with cancer with AS is necessary.

**Systematic Review Registration:**

Identifier [INPLASY CRD: 202220009].

## Introduction

With the uptrend of aging in the world, the morbidity of valvular disease in the elderly is increasing, in which AS has gradually become the most common valvular heart disease in the elderly. The main manifestations of AS are angina pectoris, syncope, dyspnea, and even sudden death. The effect of conservative treatment is not good, though it can relieve the clinical symptoms, the aortic valve function cannot recover, affecting the quality of life of patients. The results of the American population survey showed that the incidence of severe valvular disease in the elderly is 2.5%, including 13.3% in people over 75 years old. European surveys showed that the incidence of AS in the population is 4%, and 2% in the elderly population (1). In addition, not only the incidence of AS is increasing year by year, but also the prognosis is very poor. Once the symptoms or cardiac function decrease, the mortality increases sharply. If only conservative treatment is performed, the 2-year fatality rate is 50% to 60%. Therefore, active intervention is needed.

Since transcatheter aortic valve implantation (TAVI) appeared in 2002, it has become a vital treatment of choice for patients with severe AS (2, 3). TAVI is sending the artificial valve to the aortic valve area to replace the aortic valve to perform its functions. TAVI indications listed in the 2017 European Valve Management guidelines: symptomatic patients with severe AS who are not suitable for surgery (I, B); or patients with higher surgical risk are defined as STS score or Euro SCORE II ≥4%, or other risk factors, such as weakness, porcelain aorta, and chest radiotherapy, especially suitable for elderly patients with femoral artery approach (I, B). The indications for TAVI listed in the 2017 American Valve Management guidelines are symptomatic in severe patients with AS with surgical taboos or high risk and expected survival of more than 12 months (I, A); surgical risk severe AS patients (II, a).

The TAVI has quickly developed all over the world because of its small trauma and rapid recovery. At present, more than 300,000 cases have been completed in more than 60 countries (4, 5). Among them, cancer patients with severe AS become a special group of valvular disease because of tumor recurrence, metastasis, and other characteristics. However, related research on the clinical efficacy and safety of TAVI in patients with cancer with severe AS is limited and the conclusion is still controversial. Therefore, the purpose of this study is to systematically evaluate the early and medium-term clinical efficacy of TAVI in patients with severe AS with cancer.

## Data and Methods

### Data Sources

The Preferred Reporting Items for Systematic Reviews and Meta-Analyses Protocols (PRISMA-P) statement was followed. A comprehensive literature search was performed through the PubMed, Embase, The Cochrane Library, CBM, CNKI, and Wanfang databases from their establishment to October 2021 using the following terms: “transcatheter aortic valve implantation,” “transcatheter aortic valve replacement,” “TAVI,” “TAVR,” “neoplasm,” “malignancy,” “cancer,” and “tumor” with no restrictions on language. Reference lists of reviewed articles were screened to identify further relevant studies. When outcomes reporting was incomplete, the study authors were contacted for further information.

### Study Selection

Inclusion criteria were as follows: studies performed in patients with severe AS and cancer; study design comparing patients with cancer undergoing TAVI to patients without cancer undergoing TAVI; reporting the 30-day, 1-year, and late mortality. In the meta-analysis, we included patients with an active history of cancer.

### Eligibility Criteria

All studies were included based on the following inclusion criteria: (1) the study enrolled patients with AS with cancer; (2) the study intervention was TAVI with no restrictions on the valve style (balloon- or self-expandable valve) or delivery route; (3) the study compared clinical outcomes of patients with cancer to patients without cancer undergoing TAVI; (4) the study design was randomized controlled trials (RCT) or cohort studies.

Studies will be excluded if one of the following conditions is met: (1) the type of study was case-control studies, case reports, conference abstracts, reviews, comments, or editorials were excluded; and (2) a significant amount of research data was missing or not available.

### Study Selection and Data Extraction

The first author (YS) and the second author (YW) independently screened titles and abstracts of all identified records to exclude unrelated studies based on inclusion/exclusion criteria. After that, relevant studies and full articles were reviewed to further determine their suitability. Disagreements were resolved by discussions with a third reviewer (ZW) or by consensus.

### Clinical Endpoints

The primary outcome is all-cause mortality in 30-days, 1-year, and late mortality. The second outcome included myocardial infarction (MI), stroke, bleeding events, major or minor vascular complications, new permanent pacemaker implantation, acute kidney injury (AKI), and device success.

### Risk of Bias and Statistical Analysis

The Cochrane Collaboration's tool for assessing the risk of bias was utilized to assess the risk of bias in RCTs, including: (1) sequence generation; (2) allocation concealment; (3) blinding of participants and personnel; (4) blinding of outcome assessment; (5) incomplete outcome data; (6) selective outcome reporting; and (7) other bias. Moreover, the Newcastle-Ottawa Scale (NOS) (6) was used to assess the quality of cohort studies consisting of three factors: patient selection, comparability of the study groups, and the assessment of outcomes.

Categorical variables were reported as percentages, and continuous variables were presented as the mean ± SD. We reported clinical outcomes and their respective effect size in all included studies using odds ratios (ORs), with corresponding 95% confidence intervals (CIs).

Heterogeneity assessments were performed using χ2-based Q statistics and I^2^ tests. If *P* > 0.10 and I^2^ ≤ 50%, there was no statistical heterogeneity among results; if *P* <0.10 and I^2^ > 50%, there was a considered significant heterogeneity. All the results were performed using the random effect model. Subgroup analyses were also performed to find more potential information based on a different type of event. The likelihood of publication bias was assessed directly through the funnel plots, evaluated using an Egger's test. All analyses were performed using Review Manger 5.3 and Stata 12.0 software.

## Results

### Baseline Demographic and Quality Assessment

**Figure 1:**
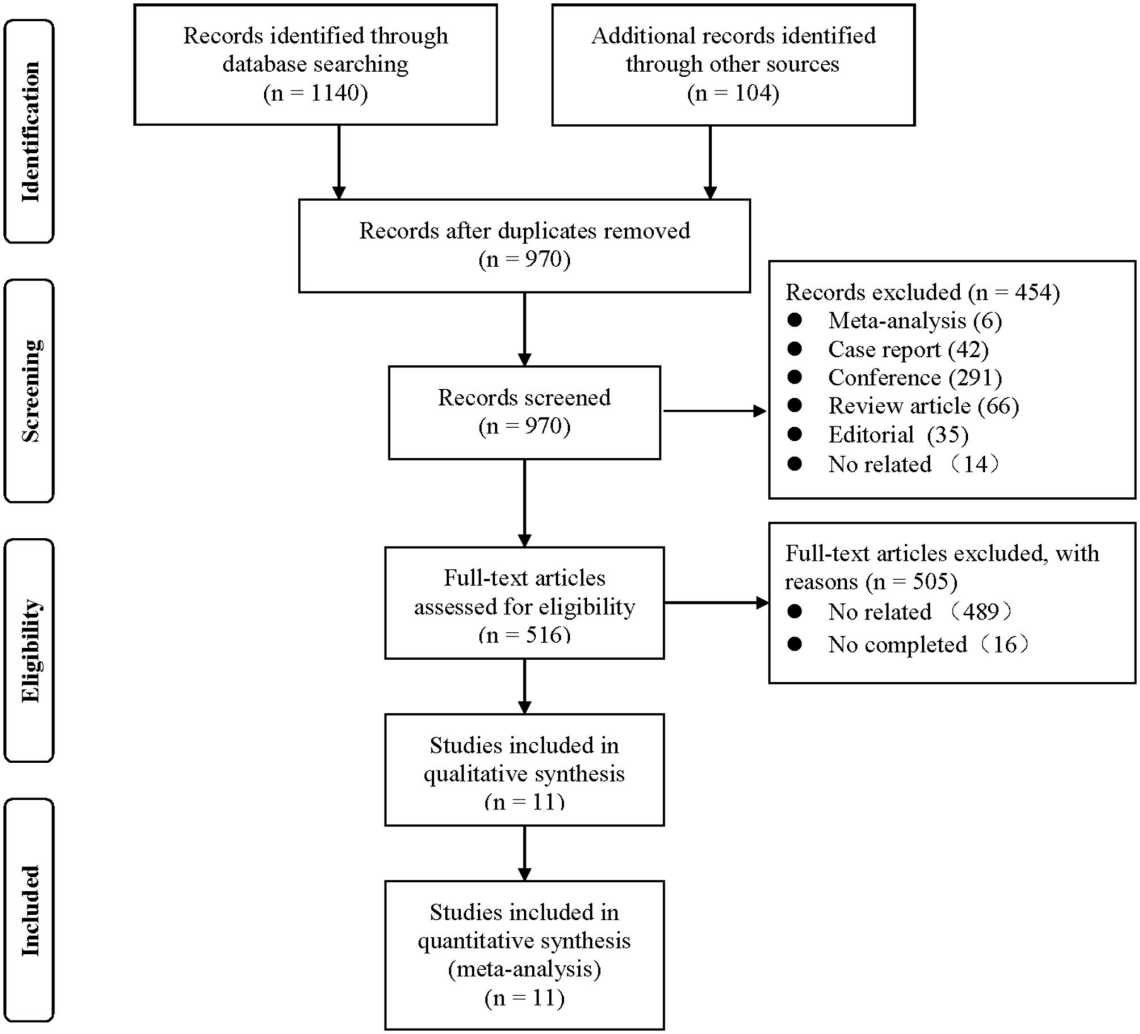
Flow diagram for study selection.

A total of 1,140 potentially eligible studies were identified in our initial search, and 11 clinical studies met the inclusion criteria (5, 7–16) (Figure 1). A total of 182,645 patients were enrolled, including 36,283 patients in the cancer group and 146,362 patients in the non-cancer group. The basic information of these studies is in Table 1. There were significant statistical differences in the mean Society of Thoracic Surgeons score (STS score) [*WMD* = −0.76, 95%*CI* (−1.14, −0.37), *I*^2^= 70%, *P* = 0.0001] and logistic European System for Cardiac Operative Risk Evaluation II (logistic Euro SCORE II) [*WMD* = −0.95, 95%*CI* (−1.25, −0.65), *I*^2^= 0, *P* <0.00001] between two groups.

**Table 1:** Characteristics of the studies included in this meta-analysis.

**NO**	**Reference**	**Year**	**Type of research**	**Samples** **(E /C)**	**Characteristics(E/C)**				**Medical history(E/C)**					**Inspection report(E/C)**			**NOS**
					**Age(year)**	**Male(%)**	**BMI(Kg/m2)**	**Euro** **ScoreII(%)**	**Hypertension(%)**	**DM(%)**	**MI(%)**	**NYHA III,IV(%)**	**PAD(%)**	**STS score(%)**	**Valvular area(cm^**2**^)**	**LVEF(%)**	
1	Watanabe et al. (16)	2016	Cohort studies	47/702	83.0 ± 5.2/85.0 ± 4.4	45.0/33.0	23.6 ± 3.8/21.7 ± 3.6	3.1 ± 2.4/3.9 ± 2.8	75.0/75.6	30.0/25.0	11.0/8.0	40.0/48.0	23.0/15.0	5.4 ± 3.0/7.0 ± 3.6	0.65 ± 0.1/0.62 ± 0.2	65.9 ± 9.2/65.0 ± 7.8	8
2	Berkovitch et al. (7)	2018	Cohort studies	91/386	79.4 ± 8.6/81.8 ± 7.0	52.0/52.0	NR	4.5 ± 4.8/5.4 ± 5.9	82.0/85.0	34.0/40.0	NR	NR	NR	4.6 ± 3.0/5.7 ± 3.9	NR	NR	7
3	Mangner et al. (13)	2018	Cohort studies	350/1471	80.3 ± 5.7/81.0 ± 5.2	47.1/42.7	27.1 ± 4.9/27.4 ± 5.0	NR	92.6/93.6	40.6/43.6	12.3/12.0	78.3/77.1	10.3/11.7	6.4 ± 4.8/6.7 ± 4.8	0.6 ± 0.2/0.7 ± 0.2	58.4 ± 13.6/58.0 ± 14.8	8
4	Landes et al. (5)	2019	Cohort studies	222/2522	78.8 ± 7.5 /81.3 ± 7.1	62.1/45.0	26.6 ± 4.8/28.0 ± 5.0	4.2 ± 3.2/5.4 ± 4.4	76.0/92.0	28.0/36.0	13.0/9.0	76.0/83.0	16.0/14.0	4.9 ± 3.4/6.2 ± 4.4	0.72 ± 0.22/0.65 ± 0.20	56.0 ± 14.0/56.0 ± 8.0	8
5	Tabata et al. (15)	2019	Cohort studies	240/964	80.5 ± 5.9/81.0 ± 6.3	62.5/48.5	26.4 ± 5.1/27.0 ± 6.7	6.2 ± 5.7/6.8 ± 6.5	84.2/86.5	25.4/28.4	14.2/12.4	90.3/92.3	32.9/34.6	5.1 ± 4.1/5.6 ± 5.2	0.73 ± 0.16/0.72 ± 0.17	NR	8
6	Biancari et al. (8)	2020	Cohort studies	417/1713	80.6 ± 6.6/81.4 ± 6.6	48.9/44.0	NR	NR	NR	22.8/29.8	1.9/2.4	NR	NR	4.4 ± 3.2/4.6 ± 3.3	NR	NR	7
7	Grant et al. (9)	2020	Cohort studies	23670/ 99400	81.1 ± 7.9/80.1 ± 6.7	56.7/52.9	NR	NR	81.1/79.6	31.6/36.7	NR	NR	NR	NR	NR	NR	8
8	Guha et al. (10)	2020	Cohort studies	10670/ 36625	81.1 ± 0.2/80.8 ± 0.1	57.2/52.6	NR	NR	83.5/83.8	38.0/41.5	14.0/13.4	NR	NR	NR	NR	NR	7
9	Lind et al. (12)	2020	Cohort studies	249/839	81.1 ± 5.9/81.4 ± 5.4	50.6/45.5	NR	NR	94.0/94.7	33.7/34.6	7.2/6.6	85.1/89.0	17.7/20.2	5.1 ± 1.9/6.0 ± 2.4	NR	50.6 ± 11.3/51.3 ± 11.1	8
10	Tabata et al. (14)	2020	Cohort studies	298/1270	80.8 ± 5.8/81.1 ± 6.7	60.7/47.5	26.2 ± 5.0/27.0 ± 6.5	6.2 ± 5.7/6.8 ± 6.3	NR	25.0/28.7	12.3/11.9	NR	NR	5.4 ± 4.2/5.8 ± 5.2	0.73 ± 0.16/0.72 ± 0.17	NR	7
11	Karaduman et al. (11)	2021	Cohort studies	36/514	74.6 ± 6.5/77.8 ± 8.0	30.6/43.0	25.0 ± 3.9/27.9 ± 6.2	7.4 ± 4.9/9.1 ± 5.8	75.0/82.6	19.4/30.2	NR	58.3/72.4	NR	4.8 ± 3.2/6.1 ± 3.5	NR	NR	7

*E, Experiment group; C,control group; E/C,%, proportion; BMI, Body Mass Index; Euro score, Logistic European score; DM, Diabetes Mellitus;PAD, Peripheral Artery Disease; MI,Myocardial Infarction; LVEF,Left Ventricular Ejection Fraction; NOS, Newcastle-Ottawa Quality Assessment Scale*.

### Clinical Outcomes

#### All-Cause Mortality

**Figure 2:**
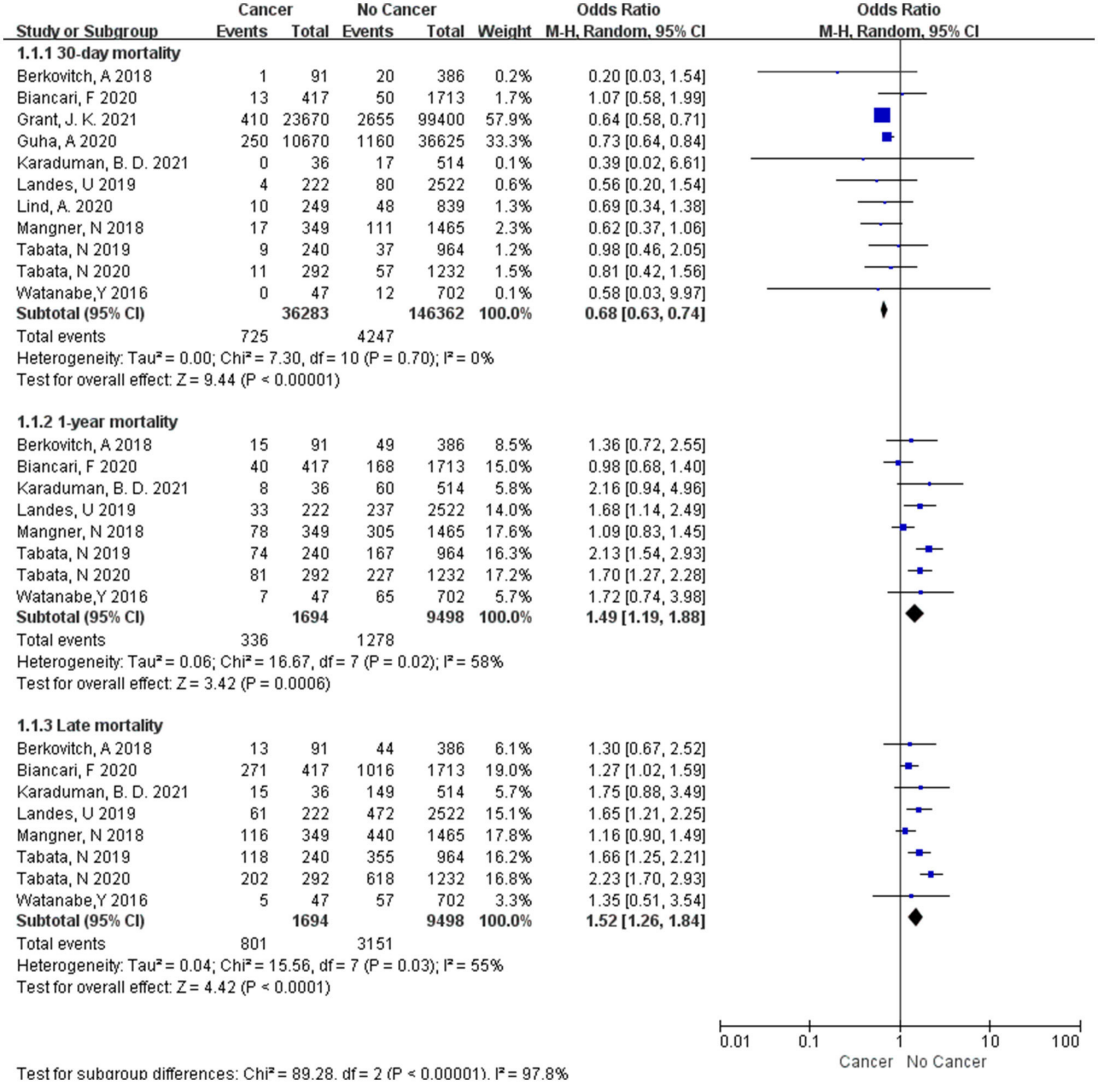
The forest plot of all-cause mortality.

For all-cause mortality, subgroup analysis of included studies illustrated that there were significant differences among them. At 30-day mortality, 11 studies were enrolled (5, 7–16) and the random effect model showed that the cancer group had a significantly lower all-cause mortality than the non-cancer group [*OR* = 0.68, 95%*CI* (0.63, 0.74), *I*^2^= 0, *P* < in 0.00001]. However, cancer group had higher mortality than non-cancer group at 1-year (5, 7, 8, 11, 13–16) [*OR*=1.49, 95%*CI* (1.19,1.88), *I*^2^= 58%, *P* = 0.0006] and late (5, 7, 8, 11, 13–16) [*OR*=1.52, 95%*CI* (1.26,1.84), *I*^2^= 55%, *P* <0.0001] (Figure 2).

#### Cardiovascular Mortality

There was no significant statistical difference in cardiovascular mortality [*OR*=1, 95%*CI* (0.83, 1.19), *I*^2^= 2%, *P* = 0.96] between the two groups.

#### Stroke

**Figure 3:**
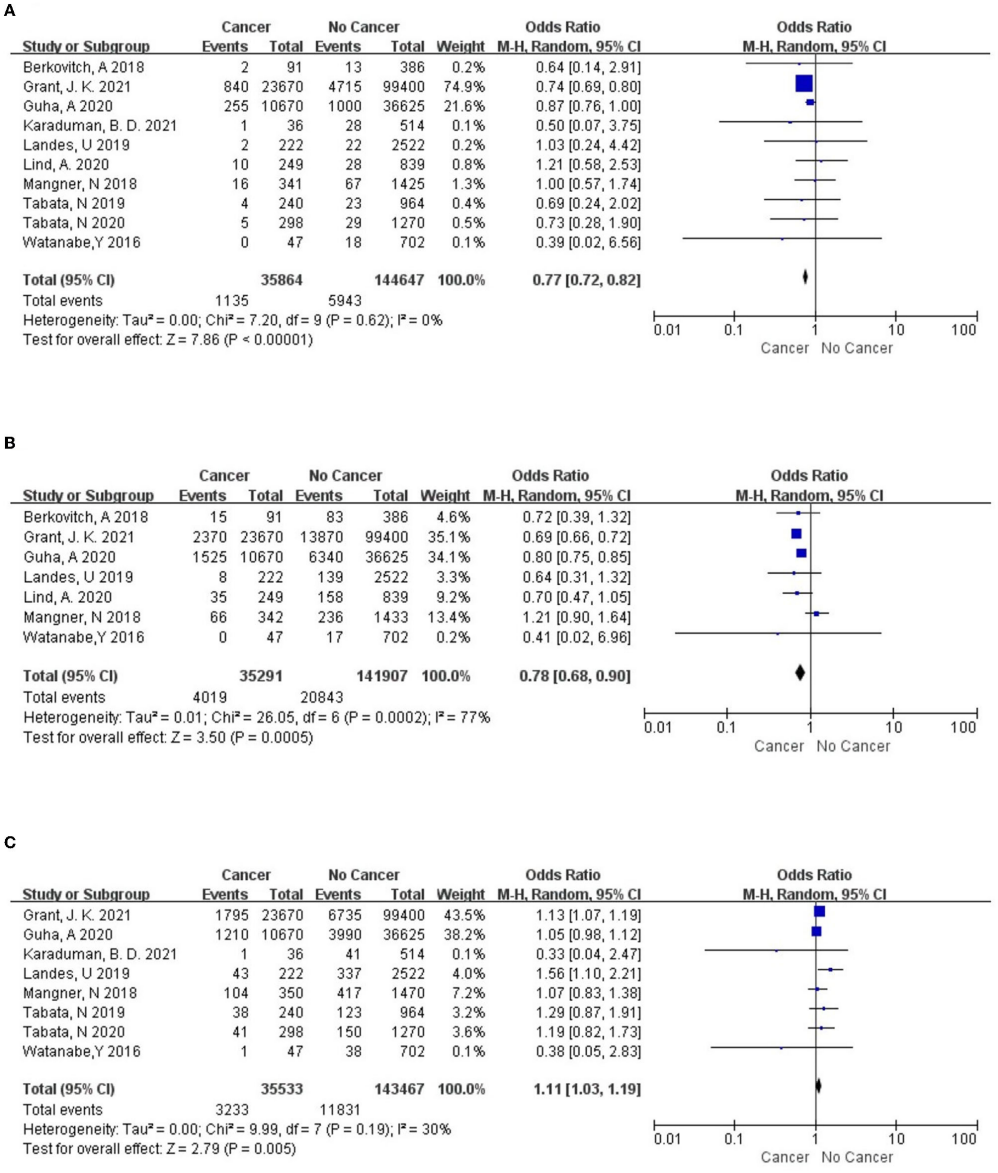
The forest plots of **(A)** stroke, **(B)** AKI and **(C)** new permanent pacemaker.

There were 10 studies (5, 7, 9–16) included and the meta-analysis showed that the patients with cancer were associated with a significantly lower rate of stroke than the non-cancer group [*OR* = 0.77, 95%*CI* (0.72, 0.82), *I*^2^= 0, *P* <0.00001] (Figure 3A).

#### Acute Kidney Injury

There were 7 studies (5, 7, 9, 10, 12, 13, 16) included and the meta-analysis showed that the patients with cancer were associated with a significantly lower rate of acute kidney injury (AKI) than the non-cancer group [*OR* = 0.78, 95%*CI* (0.68, 0.90), *I*^2^= 77%, *P* = 0.0005] (Figure 3B).

#### New Permanent Pacemaker

There were 8 studies (5, 9–11, 13–16) included and the meta-analysis showed that the patients with cancer were associated with a significantly higher success rate of new permanent pacemakers than the non-cancer group [*OR* = 1.11, 95%*CI* (1.03, 1.19), *I*^2^= 30%, *P* = 0.005] (Figure 3C).

#### Other Clinical Outcomes

There were no differences in any bleeding events [*OR* = 1.13, 95%*CI* (0.82, 1.56), *I*^2^= 84%, *P* = 0.45], device success [*OR* = 1.14, 95%*CI* (0.63, 2.08), *I*^2^= 56%, *P* = 0.66], myocardial infarction [*OR* = 0.92, 95% *CI* (0.30, 2.86), *I*^2^= 57%, *P* = 0.88], major vascular complications [*OR* = 1.16, 95%*CI* (0.76, 1.78), *I*^2^= 14%, *P* = 0.48], and minor vascular complications [*OR* = 0.72, 95%*CI* (0.35, 1,48), *I*^2^= 76%, *P* = 0.38] between two groups.

### Publication Bias

**Figure 4:**
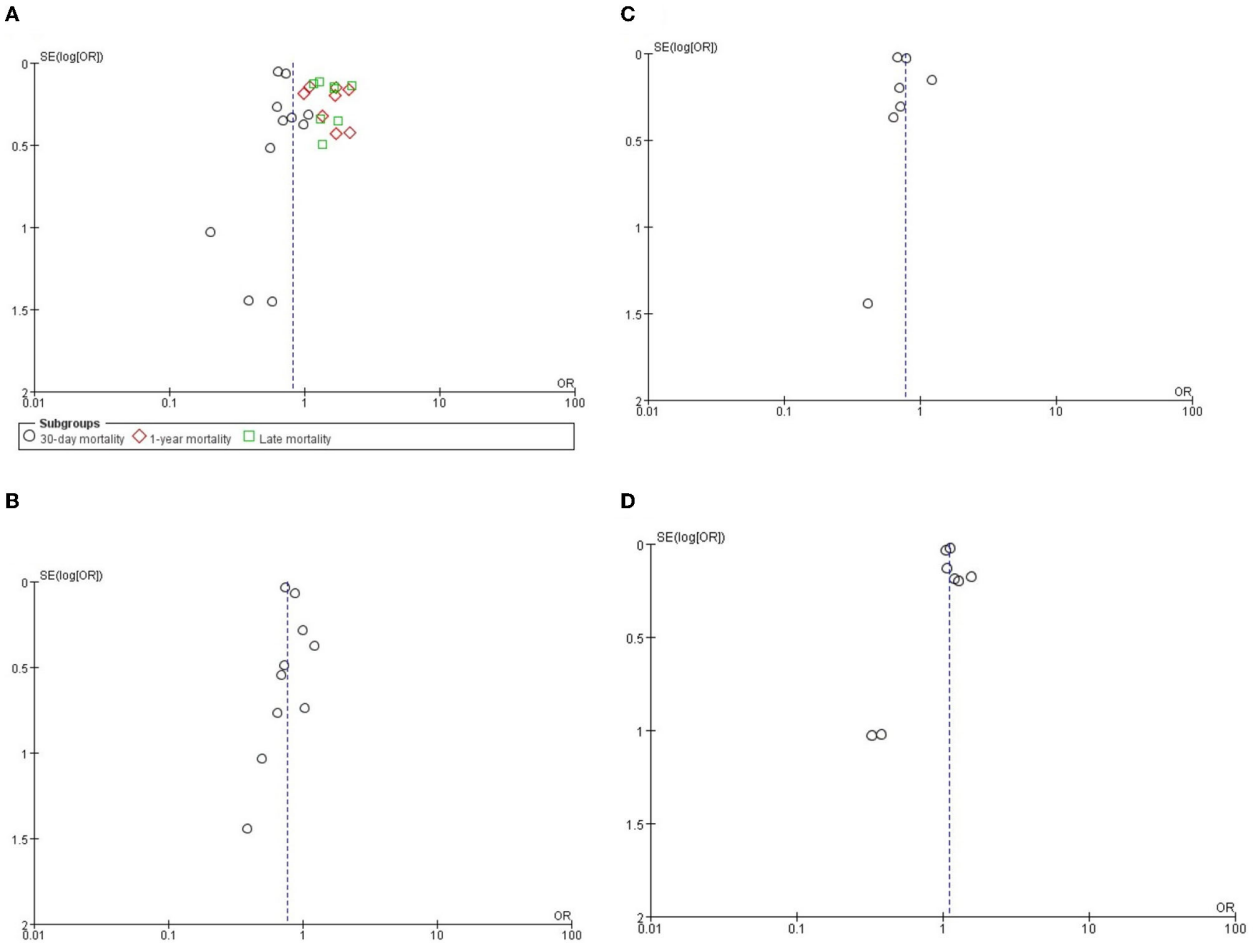
The funnel plots of **(A)** all-cause mortality, **(B)** stroke, **(C)** AKI and **(D)** new permanent pacemaker.

The funnel plot analysis and the Egger's test were used to examine the publication bias of included studies. Funnel plot analysis of all results did not show significant asymmetry. The Egger's test showed no significant publication bias in 30-day all-cause mortality (*P* = 0.819), 1-year all-cause mortality (*P* = 0.668), late and all-cause mortality (*P* = 0.806), stroke (*P* = 0.509), new permanent pacemaker implantation (*P* = 0.991), and AKI (*P* = 0.589) (Figure 4).

## Discussion

Patients with severe AS with tumors are a special group of valvular diseases (2, 3). The choice of intervention for AS is a matter of concern, because of their operation or drug intolerance, which will affect the choice of best anti-tumor therapy (5). The European Society of Cardiology (European Society of Cardiology, ESC) proposed that we can release the left heart failure caused by antineoplastic therapy by reducing the afterload of the left ventricle (17); while for AS, the afterload can be effectively reduced only through aortic valve intervention. The main clinical intervention methods for aortic valve include balloon valvuloplasty, aortic valve replacement (Surgical Aortic Valve Replacement, SAVR), and TAVI. It has been proved that balloon valvuloplasty cannot improve the survival rate of patients with AS, and has many complications (18, 19). Although SAVR can improve the survival rate of patients with cancer with severe AS (20), it will have higher perioperative mortality compared to non-cancer patients with AS because of its intolerance to open surgery (21). The revolutionary innovation of TAVI provides a great opportunity for the treatment of severe AS, which may also be the best treatment for patients with AS with cancer. TAVI has the advantages of minimal trauma and rapid recovery, which not only reduces the risk of bleeding and infection after SAVR but also avoids the interruption of perioperative antineoplastic therapy (21, 22).

The purpose of this study was to compare the difference in mortality between cancer and patients without cancer with severe AS in TAVI. The results of the meta-analysis showed that there was no significant difference in the cardiovascular mortality, any bleeding events, vascular complications, and myocardial infarction between the two groups, indicating that in patients undergoing TAVI, mortality was mainly affected by non-cardiac factors (23), such as cancer progression or metastasis. Meta-analysis showed that I^2^ was >50% in 1-year and late all-cause mortality, but much <75%, while Egger test *p*-values were >0.05, which concluded that there was no significant heterogeneity. In the 30-day, the all-cause mortality in the cancer group was lower than the non-cancer group, while in the 1-year and late all-cause mortality, the mortality in the cancer group was higher than that. Maybe in short-term treatment, TAVI relieves patients' cardiac symptoms and plays a positive role in anti-tumor treatment (24), so the short-term survival rate is increased. In addition to this, the 2017 American Valve Management guidelines state that the indications for TAVI include a life expectancy of more than 12 months after treatment to correct AS (25). Patients with cancer who choose to undergo TAVI are generally younger and have a lower risk than patients without cancer, and they also have a higher survival rate in the short term. But compared with patients without cancer, even though the patients in the cancer group are younger and have lower STS scores, the long-term survival rate decreases due to the continuous influence of tumor factors (tumor progression, metastasis, recurrence, etc.).

This study also found that in the complications after TAVI, there were significant differences in the incidence of stroke, acute kidney injury, and new permanent pacemaker. The meta-analysis showed that in the cancer group, there was a lower rate of stroke and AKI than in the non-cancer group. Stroke is a common complication after TAVI and can be classified as perioperative (within 30 days after TAVI or during hospitalization), early period (between 30 days and 1 year after TAVI), and late period (more than 1 year) depending on the time of occurrence (26, 27). A stroke occurs in the perioperative period mainly due to debris dislodgement generated during TAVI, which includes aortic wall components, atherosclerotic tissue, and valves, and it may also be triggered by damage to the aortic wall caused by the procedure (28, 29); stroke occurs in the early and late periods mainly due to valve-related turbulence, vessel wall rupture, metal frame exposure, and other procedure-related factors (30). On the one hand, patients in the cancer group had lower STS and Euro II scores than those in the non-cancer group, we believe that patients in the oncology group had better vascular conditions than those in the non-oncology group and were less likely to have a stroke due to debris from vessel wall damage or poor valve placement. The ESC/EACTS, AHA/ACC, and ESC/EAPCI committees have not reached a consensus on the choice of anticoagulation regimen after TAVI (31–33), but they all choose the appropriate anticoagulation therapy based on clinical experience and the patient's actual situation. Although patients in the cancer group are more likely to have hypercoagulable blood due to their tumors, routine anticoagulation after TAVI can reduce the risk. On the other hand, the meta-analysis showed that there is no statistically significant difference between the two groups in any bleeding events, which also demonstrates the effectiveness of either anticoagulation regimen in reducing blood hypercoagulability. While the large number of contrast media needed for an operation may cause acute renal function damage after the operation, we can see from the data that the age and STS scores of patients in the cancer group are lower than those in the non-cancer group. The lower score indicates that the patients in this group have fewer risk factors than the non-cancer group, which leads to a lower incidence of acute kidney injury after TAVI. The conduction block is also a common complication after TAVI, so 13% of patients after TAVI need permanent pacemakers to improve survival. In this study, there were statistical differences in the new permanent pacemaker implantation between the two groups. The cancer group had a higher implantation rate; however, data were collected in this meta-analysis without access to the preoperative ECG results of patients, including whether they had preoperative right bundle branch block (RBBB) or atrioventricular block (34), so we considered that the higher rate of permanent pacemaker implantation in the cancer group compared to the non-cancer group may be due to the possibility that they had a high degree of atrioventricular block or were unable to remove the temporary pacemaker after TAVI.

The strength of this meta-analysis is the inclusion of 11 articles including 182,645 patients, adequately comparing the differences between cancer and non-cancer groups in terms of various outcome indicators. This study also has the following limitations: (1) no published randomized controlled trials were included, meaning the study is only included in the cohort study for analysis, which may cause certain bias; (2) the study does not carry out a cost-benefit analysis, such as hospital stay, hospitalization costs, etc., so we cannot clarify the related economic burden of TAVI and cancer treatment; (3) due to the limitations of the follow-up time included in the study, the study only analyzed the outcome indexes in the early and medium-term by Meta, and failed to explore the longer-term prognosis of TAVI in patients with severe AS with cancer; and (4) data were collected in this meta-analysis without access to the preoperative ECG results.

## Conclusion

In conclusion, it is effective and safe to apply TAVI to the treatment of severe AS in patients with cancer, but compared with patients without cancer, the long-term mortality rate is still higher. More large samples and multicenter studies are needed in the future.

## Data Availability Statement

The original contributions presented in the study are included in the article/supplementary material, further inquiries can be directed to the corresponding author.

## Author Contributions

YS provided the idea and drafted the manuscript. YW provided statistical expertise. ZW, CX, and JD contributed to the development of the selection criteria, and the risk of bias assessment strategy. TJ read, provided feedback, and approved the final manuscript. All authors contributed to the article and approved the submitted version.

## Conflict of Interest

The authors declare that the research was conducted in the absence of any commercial or financial relationships that could be construed as a potential conflict of interest.

## Publisher's Note

All claims expressed in this article are solely those of the authors and do not necessarily represent those of their affiliated organizations, or those of the publisher, the editors and the reviewers. Any product that may be evaluated in this article, or claim that may be made by its manufacturer, is not guaranteed or endorsed by the publisher.
